# Microglial inflammatory states in the progression of Alzheimer’s disease

**DOI:** 10.1002/alz.087780

**Published:** 2025-01-03

**Authors:** Matheus B Victor

**Affiliations:** ^1^ Picower Institute, MIT, Cambridge, MA USA

## Abstract

**Background:**

The ability to profile gene expression at the single‐cell resolution offers the unprecedent opportunity to define the complex cellular heterogeneity of the brain in response to pathology. However, single‐cell transcriptomics, particularly within the context of postmortem human brain samples, only provide a static snapshot of the underlying transcriptional mechanisms driving the initiation and progression of diseases.

**Method:**

To gain a more comprehensive picture of disease‐associated transcriptional programs, our research integrates single‐cell genomics with cellular reprogramming techniques for data‐driven mechanistic studies with human‐based cellular models of the brain. Here, I will discuss our recent effort in profiling the single‐cell transcriptome and epigenome of microglia isolated from human brains with varying degrees of Alzheimer’s disease (AD) pathology.

**Result:**

We generated an extensive data set of over 150,000 unique microglial transcriptomes from over 400 postmortem human brains with and without AD pathology. Integrating our big data approach with targeted CRISPR‐mediated perturbations in microglia derived from induced pluripotent stem cells (iPSCs), we defined the temporal kinetics of inflammatory transcriptional programs adopted by microglia in response to AD. Through the lens of single‐cell genomics and cellular reprogramming, I will also discuss our current work investigating the pathophysiological role of hyperexcitable circuits in the etiology of neuropsychiatric symptoms of AD.

**Conclusion:**

Our study provides essential insight into the progression of inflammatory states in microglia, providing a roadmap for therapeutics aimed at curbing neuroinflammation and microglia pathology associated with AD in a disease‐stage specific manner.

